# Content analysis of social media regarding obesity as a chronic disease

**DOI:** 10.7717/peerj-cs.1321

**Published:** 2023-04-24

**Authors:** Farah Abdul Razzak, Diane Saab, Firas Haddad, Jumana Antoun

**Affiliations:** 1 Department of Family Medicine, American University of Beirut, Beirut, Lebanon; 2 American University of Beirut, Beirut, Lebanon

**Keywords:** Obesity, Social medial content analysis, Chronic disease

## Abstract

**Background:**

Social media is an effective online communication channel. Obesity has been classified as a chronic disease; yet, social media rarely portrays it as such. This study aims to explore the perception of obesity as a chronic disease through content analysis of social media content of obesity-related health organizations and weight loss commercial applications.

**Methods:**

Using a codebook adapted from the definition of chronic disease, content analysis was conducted to evaluate a set of posts sampled from 11 health-related organizations and 10 weight loss applications Facebook and Twitter accounts. Descriptive statistics were used to assess the extent obesity was portrayed as a chronic disease.

**Results:**

A total of 8,106 posts were extracted: 3,019 posts by organizations and 5,087 by weight loss commercial applications. Only 401 (4.5%) posts/tweets were related to obesity as a chronic disease and were posted by obesity-related health organizations. Only 69 (2.0%) posts from all the organizations’ posts directly addressed the idea that obesity is a chronic disease. Almost none of the weight loss commercial apps’ social media accounts tackled any aspect of obesity as a disease. Commercial applications’ posts revolved mainly around recipes, exercise regimens, and behavioral advice, whereas organizations tackled more complications, treatment, and obesity bias.

**Conclusion:**

Using content analysis of social media content, obesity-related health organizations and weight loss applications did not emphasize obesity as a chronic disease on their social media platforms of Facebook and Twitter. Weight-loss commercial applications on social media should include more posts to modify the public’s perception regarding obesity as a disease, contributing to health promotion. Further research should explore other social media platforms and posts with specific hashtags posted by the general population.

## Introduction

Social media is a communication channel between science and the public (Hunter, 2016), where healthcare professionals can share health information with their patients. The media frames the public’s opinions and perceptions about health issues in easily comprehended and disseminated formats (Aguilar & Terán, 2016), affecting their health attitudes and behaviors (Chen et al., 2018; Depue et al., 2015; Ortiz, Smith & Coyne-Beasley, 2019). Social media serves as a form of health communication, fostering interaction and sharing, facilitating health messages, and contributing to health promotion and healthcare policymaking (Moorhead et al., 2013).

Obesity is a global public health problem, with the World Health Organization reporting a threefold increase in global prevalence since 1975 (Organization, 2021). Obesity was recognized as a medical condition as the American Medical Association (AMA) recognized it as a chronic disease requiring medical attention in 2013 (Pollack, 2013), and multiple other professional health organizations followed later (Bray et al., 2017). Since then, the public has shown some acceptance of obesity as a disease (AlArab & Antoun, 2022; Puhl & Liu, 2015), yet the adoption of this novel concept has been slow, particularly on social media.

Social media frames obesity with prominent stigmatization (Jeon et al., 2018; Stanford, Tauqeer & Kyle, 2018; Wanniarachchi et al., 2020) where people with obesity are often portrayed as unhealthy individuals who lack willpower and self-control (Lupton, 2018; Stanford, Tauqeer & Kyle, 2018; Yoo & Kim, 2012). According to recent media research, the dominant themes in obesity-related media reports focus on individual diet and physical activity choices and less frequently on the complex interactions of biology, society, and environmental factors that contribute to obesity (Chou, Prestin & Kunath, 2014; Lupton, 2018; Lydecker et al., 2016; So et al., 2016; Stanford, Tauqeer & Kyle, 2018; Yoo & Kim, 2012). While social media support groups exist (Dahl, Hales & Turner-McGrievy, 2016), they often focus on behavioral solutions. The media seems to fail to portray obesity as a multifactorial disease state. Therefore, this study aims to explore the perception of obesity as a chronic disease through content analysis of social media content of obesity-related health organizations and weight loss commercial applications.

## Materials and Methods

This cross-sectional study used content analysis of Facebook posts and tweets to identify social media communication regarding obesity as a chronic disease. The American University of Beirut granted ethical approval to carry out the study (SBS-2021-0065).

### Study setting and sample

This study evaluated a sample of Facebook posts and tweets of 11 major obesity-related health organizations and 10 commercial weight loss application accounts. The list of health organizations (Appendix A) was selected from a list of 26 organizations and events curated by the ACTION study, landmark research designed by a multi-disciplinary steering committee that comprised representatives from the Obesity Society, the Obesity Action Coalition and the Integrated Benefits Institute, and other obesity experts (Depue et al., 2015). As for commercial weight loss apps, we used Healthline lists for the best weight loss apps of 2021 (10 apps). The Healthline team manages an extensive medical network of over one hundred professionals covering more than fifty specialties, ensuring that published information is accurate and evidence-based (Wang et al., 2021). All posts and tweets were publicly accessible online, and no names were recorded.

### Data collection

General information about the Facebook or Twitter account was collected. For the organizations, the following data were collected: the aim of the organization; whether it is local, international, or regional; the number of its members; the number of followers, and the total number of posts. As for weight loss applications, data collected included: focus of the app, whether the app was paid or free, the total number of posts, and total number of followers. Appendix A shows the characteristics of the organization or app’s social media accounts.

All posts (Facebook) and tweets (Twitter) between Aug 1, 2020, and Jan 31, 2021, were extracted manually to an excel document that is password-protected and shared among the research team. Duplicates of the same post or tweet within the same social media platform or across the two platforms were included. If the post was a retweet or share of another Facebook post, only the text posted by the concerned organization was included; any linked images, videos, or websites were excluded. Written posts other than English were excluded. In the excel sheet, each organization or app was given a code; therefore, the posts were deidentified. From each post or tweet, we recorded the date, the full post/tweet, and the code of the organization/weight-loss app.

### Codebook development

The research team developed a codebook to interpret whether the meaning of the post/tweet text is related to the concept of obesity as a chronic disease. The initial codebook was derived from the elements of obesity as a chronic disease: chronicity, multifactorial causes, risk factors, symptoms, complications, prevention, and treatment. Using the initial codebook, a deductive approach was used to code 5–10% of the posts and tweets. Two investigators (DH and FH) coded each post or tweet independently, with the possibility to code a post with more than one code. The investigators used an inductive method to identify additional codes based on the data throughout this round. The study team collaboratively developed an updated final codebook applied to the data (Table 1).

**Table 1:** Codebook.

Main categories	Code
The target audience of the message	Health care professionals; public
Obesity is a chronic disease	Chronic nature
	Disease
	Relapsing nature
Causes of obesity	Multi-causal disease
	Combination of inherited factors, environment, personal diet, and exercise choices
	Genetic
	Behavioral
	Metabolic
	Mental
	Hormonal
	Fat cells
	Pathophysiology process (leptin, ghrelin, *etc*.)
	Environmental (fast food, unprocessed food)
	Microbiome
Risk factors to develop obesity as a disease	Family inheritance and influences
	Lifestyle choices
	Certain diseases and medications
	Social and economic issues
	Age
	Lack of sleep
	Stress
Symptoms	---
Complications or obesity-associated diseases	Diabetes, cancer, HTN, cardiovascular disease, sleep apnea; osteoarthritis, gall stone disease
Quality of life	---
Prevention	**Exercise** Eating plan
Diagnosis	Health history
	Physical exam
	BMI
	Waist circumference
	Blood tests
Treatment	Dietary changes
	Exercise and activity
	Behavior changes (self-monitoring, cognitive behavioral therapy, *etc*.)
	Weight loss medications
	Endoscopic procedures for weight loss and weight loss surgery
	Other treatments (vagal nerve block)
	Clinical trials
	Alternative medicine (herbal remedies, vitamins and minerals, Mind-body therapies—such as acupuncture, mindfulness meditation and yoga)
	Coping and support
Weight bias	---
Irrelevant post	

### Data analysis

Content analysis was used to interpret the meaning of the posts’ texts. The unit of analysis was the whole post or tweet. Two investigators independently identified the category of the posts/tweets based on the codebook. First, the post/tweet was identified as related to obesity as a chronic disease or irrelevant. If it was related to obesity as a chronic disease, the post’s category was identified using the codebook. Any discrimination was resolved by the third investigator (JA) after a discussion with the team. The excel sheet data was imported into IBM SPSS statistical package, version 26, for data analysis. Descriptive analysis was performed to quantify the number of codes and categories as percentages of the total posts. Sub-analysis was performed based on the health organizations and apps.

## Results

**Figure 1:**
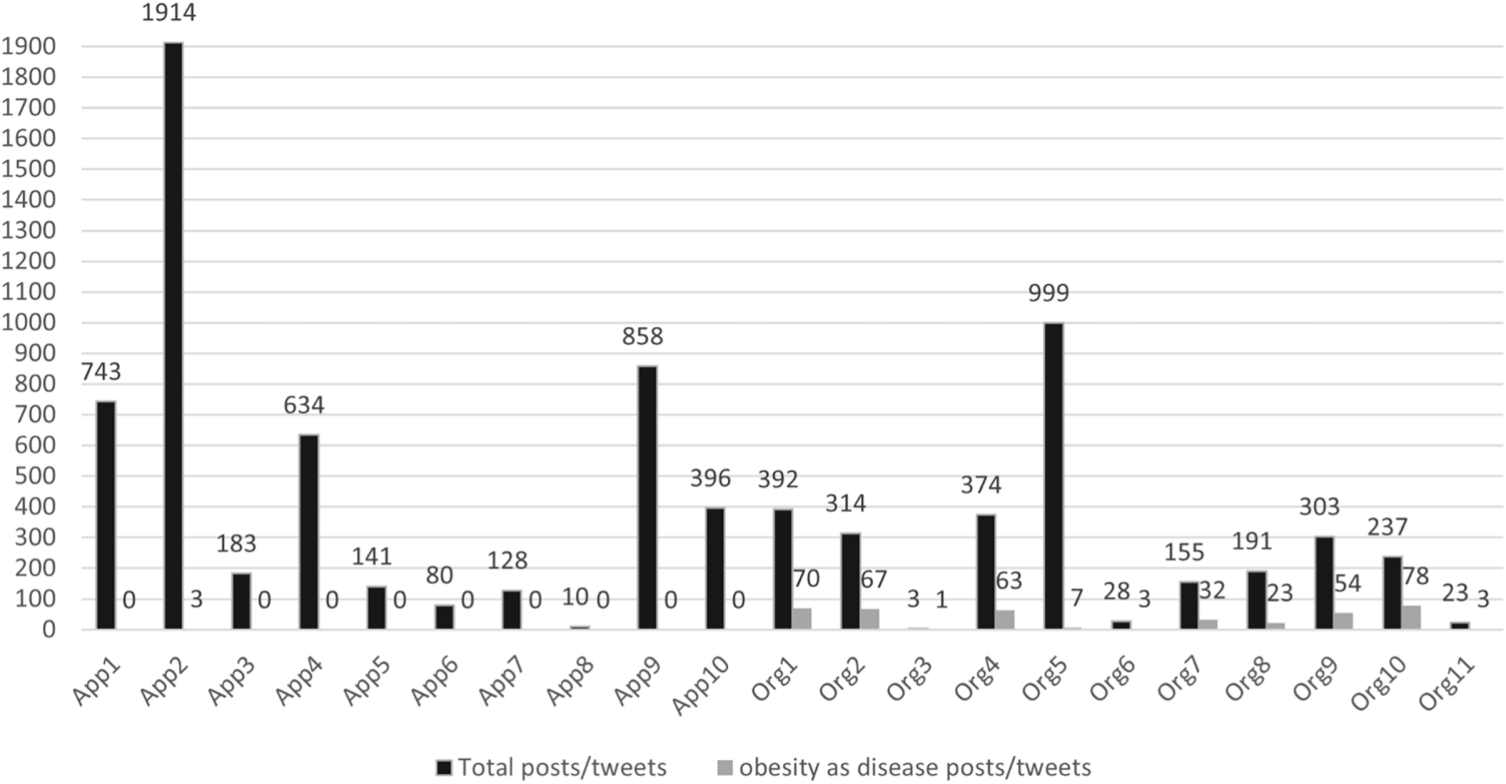
Posts/tweets of health-care organizations and commercial weight loss applications’ social media accounts.

A total of 8,106 posts were extracted, where 3,019 were posted by obesity-related organizations and 5,087 were posted by weight-loss commercial apps. Most of the posts were on Facebook (5,121), while the rest were on Twitter (2,985). On average, there were 26.5 ± 8.4 posts per day on Facebook and 16.2 ± 16.5 on Twitter in 6 months, from Aug 1, 2020, to Jan 31, 2021. Daily, organizations posted an average of 1.9 ± 7.2 on Facebook and 1.8 ± 1.5 on Twitter. Weight-loss commercial app social media accounts had, on average, 2.3 ±1.7 posts per day and 4.6 ± 9.3 on Twitter. Figure 1 shows the total posts and obesity-related posts by each app and organization account.

Only 4.5% (401 posts/tweets) were related to obesity as a chronic disease and were posted by obesity-related health organizations. Only 69 (2.0%) posts from all the organizations’ posts clearly stated that obesity is a chronic disease. Almost 99.9% of the app-weight loss social media accounts posted on exercise, recipes, nutrition, and behavioral advice, and almost none tackled any aspects of obesity as a disease. Table 2 shows the percentage of the codes by organizations and weight-loss app social media accounts. Obesity-related complications were primarily coded among health organizations’ social media posts (*n* = 122, 4.0%), and few posts targeted prevention, obesity causes, and risk factors. Table 3 shows the comments or phrases used to frame obesity as a chronic disease.

**Table 2:** Distribution of the codes assigned to the posts.

	Organization *N* = 3,019	App *N* = 5,087
Chronic disease	59 (2.0)	0 (0)
Obesity causes	31 (1.0)	1 (0)
Obesity risk factors	32 (1.0)	2 (0)
Obesity complications	122 (4.0)	0 (0)
Obesity and quality of life	2 (0.1)	0 (0)
Obesity prevention	7 (0.2)	0 (0)
Obesity treatment (medication or surgery)	82 (2.7)	9 (0)
Obesity bias	76 (2.5)	0 (0)

**Table 3:** List of comments regarding obesity as chronic disease.

“Obesity as a disease”.
“Obesity is not only a complex disease but also a complex topic”.
“Obesity should be treated just like any other disease in the media”.
Americans are beginning to understand obesity more as a medical condition and less as a personal fault
Chronic relapsing disease
Complex disease
Complicated disease
Raise awareness of the disease of obesity
Refocus the perception of obesity and change the perception of individuals affected by this chronic disease
Right to evidence-based treatment for the disease of obesity
Obesity management must be based on the principles of chronic disease management
Perceive obesity in the same light as any other disease

## Discussion

This study examined the content of Facebook and Twitter posts to assess whether significant obesity health-related organizations and weight loss commercial applications frame obesity as a chronic disease. Unsurprisingly, obesity as a disease was almost non-existent in 8,106 posts over 6 months. Only 2% of obesity health organization-related posts included obesity as a disease, and it was almost non-existent in social media accounts of weight loss applications. Our findings align with what is known about obesity framing in social media. Social media platforms (Facebook, Twitter, Youtube) and traditional sources like newspapers display an unbalanced emphasis on the triad of obesity, diet, and physical activity (Atanasova & Koteyko, 2017; Chou, Prestin & Kunath, 2014; Hussin, Frazier & Thompson, 2011; Lupton, 2018; Lydecker et al., 2016; Sandberg, 2007; So et al., 2016; Stanford, Tauqeer & Kyle, 2018; Yoo & Kim, 2012). Social media is an example of a powerful tool that the general public can easily access and use to participate in change and spread misinformation (Valenzuela et al., 2019). So et al. (2016) analyzed 121,268 tweets about obesity between Jan 2012 and March 2012. Obesity-related tweets addressing individual-level causes for obesity received more retweets and shares than tweets that looked at societal issues like the availability of cheap or unhealthy food (So et al., 2016). Similarly, reviews of widespread obesity YouTube videos concluded that most depictions of obesity were linked to personal factors (Hussin, Frazier & Thompson, 2011; Yoo & Kim, 2012).

The media bias for individual framings could be multifactorial. It may be deep-rooted in a desire to please the readers. The public perception of obesity as a disease is still slow (Puhl & Liu, 2015), leading us to believe that the media prefers familiar and well-known topics that appeal to the general public. In addition, the public prefers reading about concrete situations in which they can identify themselves rather than the more abstract and didactic aspects of obesity (Bastian, 2011). This could explain why social media pays so little attention to obesity as a disease. Furthermore, the authors of this study theorize that weight loss applications are designed to provide subscribers with better diet plans and rigorous weight loss exercises. Hence, it would be more in alignment with their purpose of creation to have their social media platforms focus on the former issues mentioned rather than on the management of obesity as a disease requiring follow-up with health care providers.

### Comparison with prior work

Obesity discussions differ across social media platforms. Chou, Prestin & Kunath (2014) analyzed 2.2 million posts to study weight-related social media interactions. Condescending and derogatory comments with the content of weight stigmatization flooded Twitter and Facebook, while more insensitive comments flooded blogs and forums (Chou, Prestin & Kunath, 2014). In our study, we only analyzed Facebook and Twitter content. Still, we did not record any discriminatory or weight-stigmatizing comments as the social media accounts of both the obesity health organizations and weight loss applications are professional. Yet, in our study, weight bias was discussed in only 2.5% of the obesity health organization posts. It seems their social media accounts fail to promote awareness about obesity as a disease (as previously mentioned) and other important topics such as weight bias. In addition, Chou, Prestin & Kunath (2014) also reported the impact of language usage. Using the words obese and obesity rather than fat resulted in more discussions that focus on obesity as a disease (Chou, Prestin & Kunath, 2014). Our study findings show that even when the word obese is used, and obesity is addressed as a disease, obesity health organizations discuss it superficially without going into depth. Obesity health organizations need to move towards a more proactive role in changing the perception of obesity as a disease and decreasing weight bias.

In the past few decades, social media has proven its efficacy in raising awareness and creating change in public perceptions and attitudes on various social issues such as smoking (Naslund et al., 2017; Yoo, Yang & Cho, 2016), HIV testing, and adherence to medication (Bychkov & Young, 2018; Wang et al., 2019), and most recently COVID vaccinations (Puri et al., 2020). Social media has been used for public awareness regarding the appropriate use of antibiotics (Catalan-Matamoros, Pariente & Elias-Perez, 2019). Concerning obesity, news media frames show a significant impact on weight-related attitudes. Compared with people who read fat-positive frames (that highlight obesity as uncontrollable by individual factors), people who read fat-negative frames (that highlight obesity as a controllable issue) were more likely to negatively judge obese people and support weight-based job discrimination and prejudice. Yet, adding anti-stigma messages tempered the expression of prejudice (Frederick et al., 2020). Social media is a cost-effective tool that can hold obesity support groups (Dahl, Hales & Turner-McGrievy, 2016), fight weight stigma (Zavattaro, 2021), and potentially educate the public about obesity as a disease entity. Health organizations, healthcare providers, and governments should play a key role, through social media platforms, in providing the public with evidence-based information about obesity as a complex multifactorial disease.

### Strengths and limitations

This study analyzes how social media portrays obesity as a chronic disease. It’s the result of a manual content analysis of many posts from two popular social medial platforms. It adds to our understanding of the information displayed to the public regarding the obesity pandemic. However, some limitations affect the generalization of the results. First, only Facebook and Twitter posts were analyzed. Even though these are still the most popular social media platforms, they are not representative of all social media platforms. Second, because only publicly accessible posts were mined, a substantial amount of private content may be left out of the analysis. Finally, this study does not look into social media conversations. It only analyzed initial posts as we were mainly interested in exploring the information organizations and commercial apps are disseminating.

## Conclusions

Despite 10 years of announcing obesity as a chronic disease, this concept is still not well disseminated. Although health-related organizations have started to share awareness on the nature of obesity as a chronic disease, it is not yet satisfactory. As for weight loss applications, the main focus of their social media content is recipes, exercise, and advice. This underestimates the true nature of obesity and considers it a state of overeating and under-exercising. The media can play a critical role in modifying the public’s perception regarding obesity as a disease to help in its prevention and management. Stakeholders of weight loss applications have a role in educating the public on obesity as a chronic disease. Individuals struggling with weight gain may follow weight loss applications on social media more than professional-healthcare organizations. Further research should look into other social platforms alongside posts with specific hashtags posted by the general population.

## Supplemental Information

10.7717/peerj-cs.1321/supp-1 Supplemental Information 1 Raw data of rating of posts and code.

10.7717/peerj-cs.1321/supp-2 Supplemental Information 2 Characteristics of the final list of organizations and weight-loss apps selected for social media content analysis.

## Additional Information and Declarations

### Data Availability

The following information was supplied regarding data availability: The raw data are available in the Supplemental File.
