# Supplementary material for: Content analysis of social media regarding obesity as a chronic disease
Source: PeerJ Comput Sci. 2023 Apr 24;9:e1321. doi: 10.7717/peerj-cs.1321 (PMC10280592; doi:10.7717/peerj-cs.1321)
Supplement: Supplemental Information 2 [file peerj-cs-09-1321-s002.docx]

**Appendix A:** Characteristics of the final list of organizations and weight-loss apps selected for social media content analysis.

|  | **Characteristics** | | | | |
| --- | --- | --- | --- | --- | --- |
| **Organization** | Aim of organization | International, regional or local | Number of members | Number of followers | Total number of posts in the 6-months period interval |
| **Obesity Information resources for people with Obesity (3)** |  | | | | |
| Obesity Action Coalition (OAC) | Giving a voice to the individuals affected by the disease of obesity. | N/A | N/A | Facebook: 18,588  Twitter: 16,100 | Facebook: 235  Twitter: 158 |
| Strategies to Overcome and Prevent (STOP) Obesity Alliance | Go beyond awareness and consumer education efforts to identify and address systemic and cultural barriers t failing to adequately support individual successes | Regional | N/A | Facebook: 2,500  Twitter: 2,831 | Facebook: 270  Twitter: 2 |
| National Obesity Care Week (NOCW) | Awareness campaign week aimed to change how we care about obesity by providing science and clinically-based education on obesity and advocating for access to affordable and comprehensive care. | N/A | N/A | Twitter:  1,976 | Twitter: 0 |
| **Obesity Health Organizations (8)** |  | | | | |
| The Obesity Society (TOS) | advancing the science-based understanding of the causes, consequences, prevention and treatment of obesity. | International | N/A | Facebook: 40,197 | Facebook:  107 |
| Academy of Nutrition and Dietetics (AND) | The Academy of Nutrition and Dietetics is the world's largest organization of food and nutrition professionals. | International | N/A | Facebook:  72,693  Twitter: 7,693 | Facebook: 675  Twitter: 319 |
| American Board of Obesity Medicine (ABOM) | Raising awareness about the field of obesity medicine and the opportunity for certification. | Regional | 5,242 (in US and Canada) | Facebook:  3,029  Twitter: 2,115 | Facebook: 16  Twitter: 12 |
| American Society for Metabolic and Bariatric Surgery (ASMBS) | The vision of the Society is to improve public health and well-being by lessening the burden of the disease of obesity and related diseases throughout the world. | Local (US) | 4000+ | Facebook: 9,260 | Facebook: 75 |
| Canadian Obesity Network (CON) | Canada’s premiere registered obesity charity dedicated to improving the lives of Canadians affected by obesity through the advancement of anti-discrimination, prevention, and treatment efforts. | Local (Canada) | N/A | Facebook: 29,397  Twitter: 21,900 | Facebook: 209  Twitter: 80 |
| European Association for the Study of Obesity (EASO) | EASO - European Association for the Study of Obesity is the leading voice of obesity science, medicine, and community in Europe, representing scientists, health care professionals, physicians, public health experts and patients. | Regional (Europe) | 36 nations | Facebook: 2,509  Twitter: 12,400 | Facebook: 303  Twitter: 0 |
| Obesity Medicine Association (OMA) | Dedicated to preventing, treating, and reversing the disease of obesity. | International | N/A | Facebook: 5,117  Twitter: 3,484 | Facebook: 108  Twitter: 129 |
| World Obesity Federation (WOF) | The World Obesity Federation is dedicated to solving the international problem of obesity by uniting scientific, medical and research communities. | International | N/A | Facebook: 2,337 | Facebook: 23 |

|  | **Characteristics** | | | | | |
| --- | --- | --- | --- | --- | --- | --- |
| **Applications (10)** | Focus of the app: activity level, food type and calorie count or behavior change | Keeps track of other health conditions or not | Information shared with other users or healthcare experts | Special features of the application | Total number of posts in the 6-months period interval | Number of followers |
| Lose It! App | Counting Calories | N/A | Other users | Barcode Scanning/ Logging food by taking pics/ syncs with fitness apps/ Challenges and motivational activities | Facebook: 348  Twitter: 395 | Facebook: 165,561  Twitter: 22,800 |
| My Fitness Pal | All the above | N/A | Other users | Motivational challenges, community and support | Facebook: 653 | Facebook: 1,552,975 |
| Fitbit App | All the above | Yes | Other users | Motivational challenges/Health solutions/Success stories/Fitbit Devices/Behavior Change/Healthy Recipes/Blog categories: activity, nutrition, stress, sleep | Facebook: 59  Twitter: 114 | Facebook: 2,965,605  Twitter: 369,200 |
| WW App | Food type and calorie count | N/A | N/A | 1-on-1 Coaching | Facebook: 573  Twitter: 61 | Facebook: 2,848,516  Twitter: 310,500 |
| Noom | All the above | N/A | Other users | Motivational challenges/Community & support/ recipes | Facebook: 122 | Facebook: 1,064,366 |
| FatSecret | Food type and calorie count (diet and nutrition partner for life) | No | Other users | Motivational challenges/Community & support/Online tools | Facebook: 69  Twitter: 11 | Facebook: 28,953  Twitter: 5,663 |
| Cronometer | Food Type and Calorie Count | N/A | N/A | N/A | Facebook: 89  Twitter: 39 | Facebook: 14,672  Twitter: 1,980 |
| Fooducate | Food type and calorie count | No | Other users | N/A | Facebook: 3 | Facebook: 303 |
| SparkPeople | All the above | Yes | Other users | Diet programs/Lifestyle Changes/Support/Ability to catalog, rate, and share healthy recipes/Nutritional Information/Calorie counter/Fitness program. | Facebook: 858 | Facebook: 660,767  Twitter: 50,300 |
| MyNetDiary | Calorie Count | N/A | N/A | N/A | Facebook: 192  Twitter: 204 | Facebook: 75,991  Twitter: 5,495 |
